# An information theoretic score for learning hierarchical concepts

**DOI:** 10.3389/fncom.2023.1082502

**Published:** 2023-05-02

**Authors:** Omid Madani

**Affiliations:** Cisco Secure Workload, Cisco, San Jose, CA, United States

**Keywords:** unsupervised learning and development, concepts, information theory, hierarchy, compositionality, constructivism, perception and interpretation, symbols

## Abstract

How do humans learn the regularities of their complex noisy world in a robust manner? There is ample evidence that much of this learning and development occurs in an unsupervised fashion *via* interactions with the environment. Both the structure of the world as well as the brain appear hierarchical in a number of ways, and structured hierarchical representations offer potential benefits for efficient learning and organization of knowledge, such as concepts (patterns) sharing parts (subpatterns), and for providing a foundation for symbolic computation and language. A major question arises: what drives the processes behind acquiring such hierarchical spatiotemporal concepts? We posit that the goal of advancing one's predictions is a major driver for learning such hierarchies and introduce an information-theoretic score that shows promise in guiding the processes, and, in particular, motivating the learner to build larger concepts. We have been exploring the challenges of building an integrated learning and developing system within the framework of *prediction games*, wherein concepts serve as (1) predictors, (2) targets of prediction, and (3) building blocks for future higher-level concepts. Our current implementation works on raw text: it begins at a low level, such as characters, which are the hardwired or primitive concepts, and grows its vocabulary of networked hierarchical concepts over time. Concepts are strings or n-grams in our current realization, but we hope to relax this limitation, e.g., to a larger subclass of finite automata. After an overview of the current system, we focus on the score, named CORE. CORE is based on comparing the prediction performance of the system with a simple baseline system that is limited to predicting with the primitives. CORE incorporates a tradeoff between how strongly a concept is predicted (or how well it fits its context, i.e., nearby predicted concepts) vs. how well it matches the (ground) “reality,” i.e., the lowest level observations (the characters in the input episode). CORE is applicable to generative models such as probabilistic finite state machines (beyond strings). We highlight a few properties of CORE with examples. The learning is scalable and open-ended. For instance, thousands of concepts are learned after hundreds of thousands of episodes. We give examples of what is learned, and we also empirically compare with transformer neural networks and n-gram language models to situate the current implementation with respect to state-of-the-art and to further illustrate the similarities and differences with existing techniques. We touch on a variety of challenges and promising future directions in advancing the approach, in particular, the challenge of learning concepts with a more sophisticated structure.

“Concepts are the glue that hold our mental world together.”, G. Murphy (Murphy, 2002).

“.. to cut up each kind according to its species along its natural joints, ...”, Plato, Phaedrus.

## 1. Introduction

Concepts such as *water, chair*, and *eat* are fundamental to human intelligence: our mental model(s) of the world and our basic cognition are founded on concepts (Murphy, 2002). What is the nature of concepts, i.e., how are they computationally represented, and how can a system acquire diverse and richly interrelated concepts in an unsupervised manner, i.e., without an explicit teacher, from the low-level sensory stream? There is evidence that much learning, of numerous concepts, and how they relate and constrain one another, and ultimately a *sense* of what is probable or common in everyday experience in humans and animals, takes place without explicit teaching, achieved largely through (active) observing (Sheridan, 1973; Gopnik and Meltzoff, 1997; Rakison and Oakes, 2003; Law et al., 2011). Inspired by considerations of early human learning, and in particular perceptual and category learning, that likely continue throughout life (Gibson, 1963; Gopnik and Meltzoff, 1997; Kellman and Garrigan, 2009; Carvalho and Goldstone, 2016), here we propose and explore an approach to efficient unsupervised learning of *discrete and discernible (interpretable/structured) concepts*, in a sparse manner, in the text domain. The learning is achieved in a cumulative bottom-up fashion.

**Figure 1:**
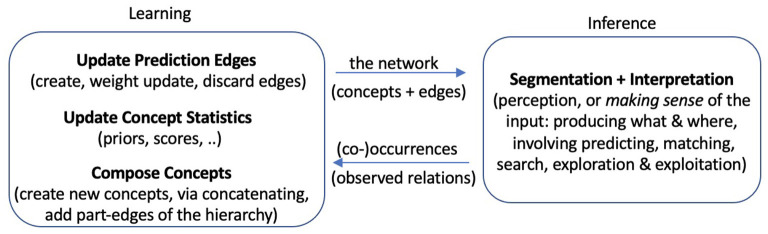
A view of the Expedition system: The two major components, inference and learning, utilize, update, and grow the network of concepts together. Several subtasks are highlighted, especially in terms of components implemented in the current system. In every (online) episode, inference uses the existing network to come up with an interpretation of the input in that episode, and the interpretation (an information-rich data structure) is given to learning components for various learning-related updates.

**Figure 2:**
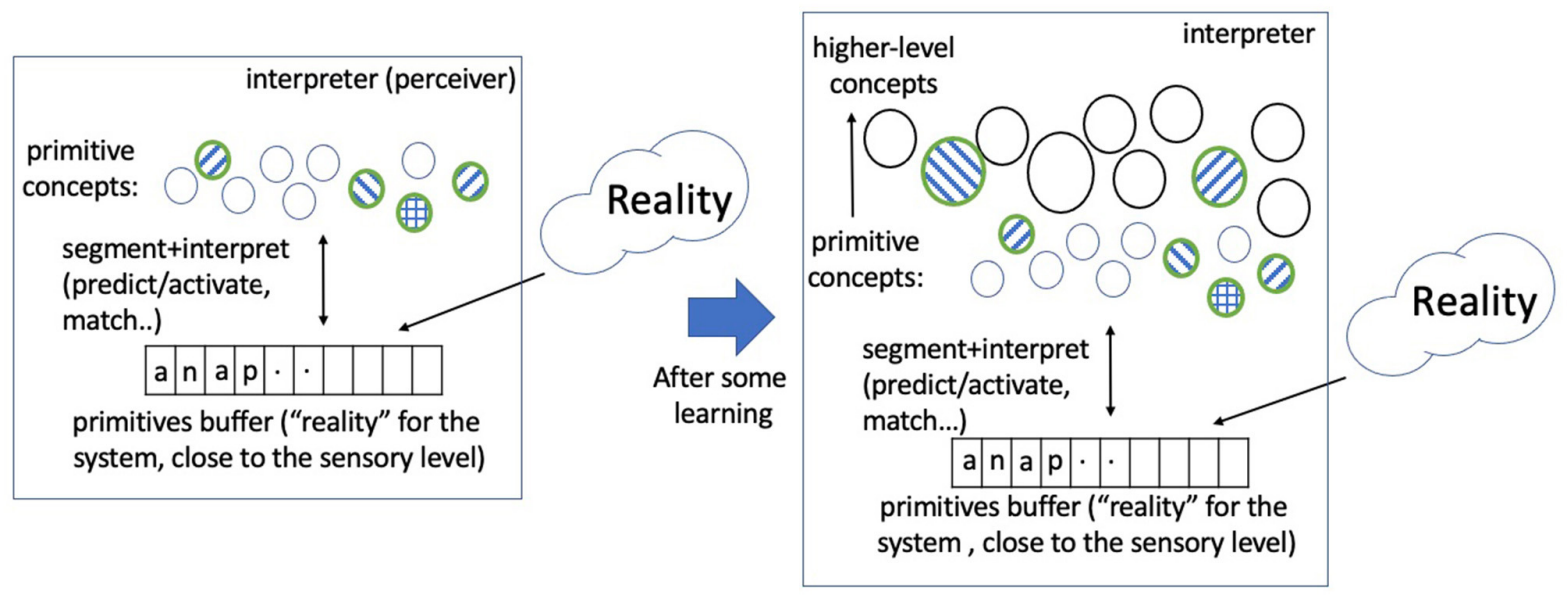
A picture of learning and development: A small portion of reality (the world, external to the system), in every episode, is mapped into a sequence of primitive concepts via sensory computations. The primitive concepts comprise a finite discrete set (an alphabet or the initial vocabulary). The system predicts and matches the concepts it currently has to the primitive sequence in an efficient manner (the interpretation process). A few concepts match and a subset is selected (the shaded ones), and learning updates occur using the observed co-occurrence relations. Over time and much learning, higher level concepts are acquired from this process, and an identical primitive sequence, presented at two different times, can lead to different sets of highest level concepts being activated due to the learning in the intervening time. The networked vocabulary of concepts grows large, but only a relatively small subset is activated in a typical episode. This study explores unsupervised learning processes that begin with a set of primitive concepts and lead to the learning and development of higher level (spatiotemporal) concepts and their rich relations.

To explore bootstrapped learning of concepts and their relations, we have developed a system, named *Expedition*, within the framework of prediction games (Madani, 2007), wherein concepts serve as (1) predictors, (2) targets of prediction, and (3) building blocks for future higher-level concepts. The Expedition system is situated in a text world in our current implementation. Text such as natural language enjoys a diversity of hierarchical regularities. A concept in this study corresponds to an n-gram or a string of consecutive characters, such as “a,” “is,” and “school.” However, a concept, as represented and used in the system, is more than just the string pattern it corresponds to: it has associations with other concepts and may have parts and may be parts of many other concepts.^1^ Expedition begins at the low level of reading single characters, which are the primitive or hardwired concepts. Each (inference+learning) episode consists of inputting a line of text, segmenting and interpreting the input in terms of a few highest level concepts, a small subset of all the concepts that currently exist in the system (inferring) and learning: updates are made to various statistics, such as prediction edge weights among the participating concepts. Interpretation is important because it determines which concepts are present in an episode, and thus how they co-occur, affecting the generation of future concepts. Periodically, the system performs offline processing, which includes building new concepts out of existing ones using the statistics accumulated over the online episodes and various other organizational and learning tasks. Therefore, the product of this learning, which is the evolving system itself, consists both of a set of structured concepts as well as the means of interpreting the environment with them, i.e., what appears in each episode. Both the edges and the nodes are necessary for interpretation. Figure 1 shows the main system components and their interaction, and Figure 2 gives a picture of the learning and development.

A major question that arises is what could drive the unsupervised learning. We posit that improving prediction of one's environment is a main driver and formalize an objective we name CORE, a combination of fit of a candidate concept to the context that contains other top-level predicted concepts (coherence), and how well the concept matches the lowest level characters. We note that when interpreting, matching the lowest level observations is often insufficient, and (the high level) context matters too. This score shows promise in guiding the interpretation of an episode as well as assessing system progress over time. Importantly, it *rewards* the system for building larger concepts and thereby growing its vocabulary of concepts, unlike related measures such as perplexity. The primary aim of this paper is to describe and motivate the information-theoretic CORE score. Another goal is to present a novel learning system, and show how it utilizes the score to use and develop concepts. We touch on a variety of challenges as we describe the various parts of the system, such as handling non-stationarity and avoiding convergence to inferior concept structures.

We expect that this overall framework for developing higher level concepts, shown in Figure 2, is not limited to text. As long as the sensory information from the external world (in various modalities) can be effectively mapped into a finite discrete vocabulary,^2^ we hope that the algorithms and the systems we explore would extend and generalize. Our work focuses on the processes that take us from the primitive space to higher level spatiotemporal concepts, or the processes behind a ***growing networked vocabulary of*** ***concepts***.

The system we have developed, with current rudimentary algorithms, is promising. When run on natural language English text with blank spaces removed, starting at the character level, we find that, after some learning, the n-grams learned, i.e., the higher level concepts, correspond to words and phrases, and the splitting of the text into concepts continues to improve with more training episodes and more time spent on inference. We also compare character-level prediction with artificial neural networks, based on the transformer architecture (Vaswani et al., 2017), and the more classic character n-gram language models (Manning and Schutze, 1999; Rosenfeld, 2000), where we obtain competitive results in predicting the first letter of a word (more suited to our current implementation of the interpretation step), even though Expedition does not optimize for predicting one character at a time. The proposed approach requires substantially less space (model size) compared with classic n-grams. The acquired patterns being explicit, compared with neural networks and traditional n-grams approaches, offers a number of other benefits, such as the possibility for more flexible inference and handling of noise (in unknown or changing future environments), learning regularities over the structure of the learned concepts (various forms of meta-learning), accelerating supervised learning tasks, and naming (a subset of) the concepts, for the purpose of communication (sharing of experience). As we explain, the benefits come at a cost, including the time and the complexity cost of interpretation in episodes, but we conjecture that, in many settings, the capabilities afforded are worth the expense: Acquiring and maintaining concepts incur costs, but they are the means for the system to untangle its world, thereby making more successful predictions, or achieving a better sense of its world. We hypothesize that acquiring such structured vocabularies is an essential step for symbolic computations.

In summary, we provide evidence that the combination of several tasks of learning and inference, with the goal of improving prediction, is a promising self-supervised framework for learning a network of structured concepts. We begin the paper with an overview of the Expedition system in the next section. We describe the network structure, i.e., the edge types and the nodes or concepts, and summarize the segmentation/interpretation process and the learning techniques used. We, then, focus on CORE in Section 3 and present several of its properties with examples. Experimental results are provided and discussed throughout these two sections. Section 4 situates this study within related research, and Section 5 concludes with a short discussion of the differentiating factors of the approach, and future directions.

## 2. Overview of the system

We describe the current implementation of the Expedition system. The system is composed mainly of a network of nodes (concepts) with a few different edge types and the operations that use the network and maintain and grow it, or the algorithms and processes. We summarize the components and provide examples instead of more formally defining the algorithms and data structures. Our prior work provides additional details (Madani, 2021a). Table 1 presents the main notation with short descriptions and pointers.

**Table 1:** Table of main notations.

** V **	**Lowest level vocabulary (an alphabet), with one-to-one mapping to primitives. |V|≈100 in most experiments**.
*T*	A text string, e.g., “ther.” A string is also shown as *c*_1_*c*_2_⋯*c*_*k*_. For “ther,” *c*_1_=“t” and *c*_4_=“r”.
*con*_*l*_(*T*)	The concept corresponding to string *T* at level *l, l*≥0 (when the concept exists), e.g., *con*_0_(“z”) is the primitive for “z” (Section 2.1.3).
Δ	Size of context (window) for prediction (default bidirectional). Δ = 3 in most experiments.
$w_{C_{1},C_{2},i}$	Prediction weight (edge) from concept $C_{1}$ to $C_{2}$, relative position *i* (*w*∈[0, 1], *i*∈{±1, ⋯ , ±Δ}). The weight is roughly a (conditional) probability (Section 2.2.6).
EMA	Exponential Moving Average, for updating edge weights (Sparse EMA), and other averages (Section 2.2.6).
*r*	Learning rate for Sparse EMA (each concept has its own rate), *r*∈[0, 1].
$\left[C_{i}\right]$	Sequence of concepts in a candidate interpretation: $C_{1},\cdots \;,C_{k}$ (Section 2.2.1)
prior(*c*)	The (lowest level) prior, or probability that character *c* is observed in a randomly picked location of a (randomly drawn input) line.
pred(C)	The prediction probability that concept C, at a specific location of an interpretation sequence, receives from context (other concepts within *j*±Δ predict) (Section 2.2.4).
CORE(C)	A combination of prediction and match scores for a concept C (in an interpretation sequence), used for guiding and scoring interpretations, and measuring system progress (Section 3).

The input to Expedition is a stream of text, where the input stream is broken into lines in our experiments. We removed blank spaces in most of our experiments, but otherwise do not do any extra processing. Thus, the line “An apple (or 2) a day!” is input into the system as “Anapple(or2)aday!.” One reason we remove blank spaces is to see whether and how well the system can learn words and phrases without the aid of separators. Does it, eventually, get the (word) boundaries right?

**Figure 3:**
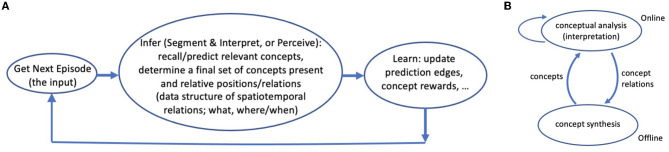
**(A)** The active perception-learning cycle: In the online phase, the Expedition system repeatedly inputs a line (some span of text), segments and interprets it (converting it to a few highest level concepts). This process creates an interpretation data structure. The system uses this information-rich data structure for (online) learning: it updates various edge weights and statistics among the active concepts present in the structure. **(B)** A simple causal view of the interaction of subsystems: offline concept synthesis changes the course of future interpretations (concept analyses), via generating new concepts, and the current interpretations affect future concept generations, through determining which (co-occurrence) relations are observed (interpretation as an internal choice or action). The self arc on interpretation means that even if concept generation is turned off, present interpretations can directly affect future interpretations, via changing the learned prediction weights (online learning).

The system alternates between two modes or phases. In the online phase, it repeatedly gets an episode (i.e., an input line) and processes it segments and interprets it, and then learns from the outcome, i.e., the resulting data structures (Figure 3 and Sections 2.2.1, 2.2.6). In the offline phase, Expedition performs other tasks that would be too expensive and wasteful to do in every online episode, such as learning new concepts (Section 2.3). The system keeps a clock, which is the number of online episodes so far. This is useful for keeping track of when a concept is first generated, used, etc.

We use a dataset of NSF abstracts in the experiments (Dua and Graff, 2017). The dataset contains approximately 120k research paper abstracts, yielding 2.5 million English text lines, over 20 million term occurrences, and just under 100 unique characters (size |V| in Table 1). Each line contains approximately 55 characters on average.^3^ All code is written in Python, and all experiments were performed on a Macbook Pro laptop. We show results and example concepts from two models which we refer to as Model3 trained to level 3 and Model4 trained to level 4 (Section 2.1.3 explains levels). Section 2.4 reports on computational cost, and Section 3.8 describes comparisons with two language modeling techniques, including artificial neural networks.

### 2.1. Network structure

The nodes in the network correspond to concepts, currently n-gram patterns. There are two main edge types: *part-related edges*, yielding a part-whole hierarchy and associations or *prediction edges*.

#### 2.1.1. Nodes

A concept corresponds to a string or a consecutive sequence of characteristics, which we may refer to as an n-gram or a string concept. A concept is also a node in a network and has different types of edges. In our implementation, both concepts and edges are object classes. Each class can have various (scalar) fields, such as a prior for concepts (probability of being observed), which can be used during segmentation. Others, such as time of creation, or first and last time seen, are useful for debugging and insights into the learning progress.

**Figure 4:**
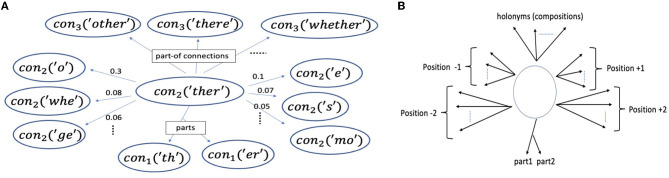
**(A)** An example learned concept, *con*_2_(“ther”) (i.e., , “ther” at level 2), from a model trained up to level 3, and a few of its prediction (three at -1 to the left, and three at +1 to the right) and part-related (vertical) edges (top 3 most frequent of its holonyms in level 3, having *con*_2_(“ther”) as a part). “o” and “whe” occur immediately before “ther” with high probability (0.3 and 0.08 respectively). **(B)** The vertical (part-related) and horizontal (prediction) edges of a generic concept (Sections 2.1.2 and 2.1.3).

Initially, before any learning has taken place, the only concepts in the system correspond to the lowest level concepts, which we refer to as primitive concepts. There is a one-to-one correspondence between the primitive concepts and the set of characters, the vocabulary V. The line of characters in each episode is readily converted to a sequence of primitives to begin the segmentation process (Section 2.2.1). Therefore, the primitives can be viewed as the interface to the external world or reality. Figure 4 shows an example concept. In our implementation, whenever a new character is first seen, a (primitive) concept object is allocated for it, so V may not be known in advance and grows over time.

#### 2.1.2. Prediction edges (associations)

Each concept keeps and updates weighted directed edges, the prediction edges to other concepts. It uses these edges to predict (Section 2.2.4). Imagining text is written and read horizontally, these connections can also be viewed as horizontal or lateral edges. Edge positions are relative to a concept. Thus, (relative) position 1, with respect to concept C, means 1 concept to the right of C, which may span several characters, and position –1 means one concept to its left. Each concept keeps edges for up to Δ positions to the left and to the right, i.e., bidirectional (although one could change to unidirectional).^4^ Thus, Δ denotes the context size (on one side). In the experiments reported in this study, Δ is set to 3 (unless specified otherwise).

Each concept keeps a separate edge-class instance for each position (with Δ = 3 and bidirectionality, we would get six relative positions). The class contains weight information as well as possibly other information such as counters and position-specific learning rates (Section 2.2.6). These weights are implemented *via* hashmaps in the edges class, and hard or soft constraints on the size of the hashmaps are imposed so that the memory consumption is kept in check, and the processing is efficient (e.g., predicting and updating times) (Madani and Huang, 2008). Examples of such edges for positions 1 and –1 are shown in Figure 4.

Let $w_{C_{1},C_{2},i}$ denote the weight of prediction edge from $C_{1}$ to $C_{2}$ for relative position *i*. A weight can be interpreted as a conditional probability, and the absence of an edge means 0 weight (Section 2.2.6). When a concept is first seen or created, it has no edges (empty edge maps).

#### 2.1.3. Part-related edges and levels of concepts

Concepts also keep track of their part-related edges: edges to their parts and edges to the concepts they are part of, i.e., their *holonyms*, which we can visualize as vertical edges, in contrast to the prediction or lateral edges (Figure 4).

In our prior work, we introduced the idea of a *layering* of and ***cloning*** of concepts so that we avoid mixing up of predictions as much as possible which we explain briefly. The pattern “a” can be a low-level concept, forming a part of many words, but it can also appear with other high-level concepts (words) as an indefinite article in English (similarly for other concepts, such as “an,” “be,” “I,” etc.). The primitive “a” should predict other primitives, while “a” at higher levels should predict other higher level concepts (Section 2.1.3 in Madani, 2021a), and we should avoid mixing these predictions. We introduced layers to help to handle such distinctions. A clone of a concept corresponds to (matches) the same pattern, but has different prediction edges (to concepts in its own layer). We note that we are working on removing this type of layering with new techniques that are based on changing (raising) learning rates. If the layering requirement is removed, several advantages include speeding up the learning and simplifying the system (note: concept structures remain hierarchical). We will use the terms “level” and “layer” interchangeably.

The primitive concepts are in layer 0. Each higher level contains the *clones* of all concepts in the layer below, and in addition, it can contain holonym concepts, which is made up of two concepts from the layer below. Thus, layer *l* contains concepts corresponding to strings that are up to 2^*l*^ characters long. A layer is added once sufficient training is performed in the current highest layer. We added layers manually in our experiments, but automating this is not difficult (see Section 2.3).

We denote the concept corresponding to a string *T* in level *i* when the concept exists by *con*_*i*_(*T*), for example, *con*_3_(“ther”). However, when clear from the context, or not important, we may not specify the level, and furthermore, we may just say the concept (corresponding to) “ther.” Higher level concepts correspond to concatenation of characters, and we also represent a string concept of size *k* by the variable C and string *c*_1_*c*_2_⋯*c*_*k*_ (where *c*_*i*_ are characters and/or primitives). Table 2 shows a number of concept sequences at different levels (Section 2.2.1), where to remove clutter, only the pattern corresponding to a concept is shown.

**Table 2:** Interpretation of a couple of example lines (episodes) *via* two models, using search width 15. The interpretation chains, i.e., concept sequences leading to the top-level sequence, covering a few lower levels are also shown. Level 1 contains up to 2-grams, level 2 up to 4-grams, and so on (Section 2.1.3). The interpretation score (average CORE, a mix of match score and received prediction probability, see Equation 1) of the top-level sequence is also shown on the left. Only the patterns corresponding to the concepts are shown to remove clutter [thus, “reg” in level 3 is actually *con*_3_(“reg”)].

**Line** = **regarding the conservation and management of these magnificent**		
**(blanks removed, input to the system is: regarding the conservation and management of these magnificent)**		
Model3,	L = 3	reg arding the conser vation and mana gement ofthese magni fice nt
11.7 CORE	L = 2	re g ar ding the con ser va tion and ma na ge ment oft hese mag ni fice nt
	L = 1	re g ar di ng t he c on se r va ti on a nd ma n a ge me nt o ft he se ma g n i fi ce nt
Model4,	L = 4	regardingthe conserv ationa ndman a gementofthe s emagni fic ent
17.3 CORE	L = 3	regard ingthe conser v ation a ndma n a gementof the s emagni fic ent
	L = 2	reg ard in gthe cons er v atio n a ndm a n a geme ntof the s ema gni fic ent
**Line** = **Commercial exploitation over the past 200 years drove**		
Model3,	L = 3	Commer cial e xploi t ationo verthe pa st t w o h undred y e ars d rove
6.3 CORE	L = 2	Comm er ci al e xplo i t ati ono ver the p a st t w o h und red y e ar s d rove
	L = 1	Co mm er c i al e xp lo i t a ti on o v er t he p a st t w o h u nd re d y e ar s d ro ve
Model4,	L = 4	Commercial e xploitatio n overthe pasttwo hundred y e ar s dro ve
11.9 CORE	L = 3	Comm ercial e xploi tatio n overthe pasttwo hundre d y e ar s dro ve
	L = 2	Comm er cial e xplo i t atio n ove rthe past two hun dre d y e ar s dro ve

Each concept keeps a list of bottom-up connections to holonyms in the next layer (its immediate holonyms) that it is a part of, as well as its clone, in the next layer. These connections are used during (bottom to top) interpretation (Section 2.2.1). The number of such connections are kept manageable. We posited that a concept need to only keep 100s to 1,000s of such connections. For instance, while the character “a” may be part of tens of thousands of words and phrases in English, the primitive that corresponds to “a” will be a part of only 10s to 100s of significant bigrams. The layering and significance tests when composing reduce the connection possibilities.

Similarly, each concept in a layer *i*≥1 keeps a list of ***top-down*** connections to its part concepts in layer *i*−1. Note that a concept corresponding to a string of *k* characters can, in principle, be split into two subconcepts (substrings) in *k* many ways. However, many such possibilities will be insignificant in the lower layer and will not be generated. However, a concept may have more than two parts, e.g., *con*_2_(“new”) can have a pair of parts (*con*_1_(“n”), *con*_1_(“ew”)), and (*con*_1_(“ne”), *con*_1_(“w”)) (the parts will always be paired).

The top-down connections are used during matching a candidate concept (holonym) during interpretation. Notably, in addition to interpretation, these vertical connections are also useful in understanding which string pattern a composition concept corresponds to.

#### 2.1.4. Special predictors

Predictors that are not themselves regular concepts, so they may not get predicted, or may not correspond to any low-level input, can be useful. We have experimented with a predictor, the *always-active* predictor, that gets updated with every position, as well as one predictor for the beginning of the input line, the *begin-buffer* predictor, and one for the end. Each level has its own set of auxiliary predictors. The always-active predictor learns to predict the prior for observing a concept at a given level,^5^ while the begin-buffer predictor learns to predict concepts that tend to appear at the beginning of a line. Note that if we set the prediction direction to left-to-right (unidirectional), the begin-buffer predictor is necessary to obtain a probability for the first concept of a sequence, in order to score the sequence (Section 2.2.5).

### 2.2. Online tasks

Every online episode consists of segmenting the input into chunks, i.e., consecutive character sequences, and interpreting the chunks in terms of existing concepts in the system. The final product of this perception process is a single data structure, an interpretation chain, that is used for updating various weights and statistics (online learning). We refer to the concepts in the final selected interpretation chain as active concepts. Next, we go over each of the two main tasks, perception and learning.

#### 2.2.1. Segmentation and interpretation

Segmentation, in general, refers to chunking (grouping) as well as separating the input bits, in our case the characters in the input line, and may or may not involve the usage of concept information. Interpretation refers to mapping chunks of input to existing concepts in the system. In our current implementation, the two tasks or processes are intertwined, and we do not make a distinction between the two segmentation and interpretation terms.^6^ This (perception) process ultimately generates a mapping from stretches of consecutive raw characters in the input to internal concepts, and, within efficiency constraints, involves bottom up activations and top-down matching processes as we describe (prediction and index look ups, probabilistic inference, search). Table 2 shows example interpretations. Note that interpretation is a kind of an internal action or decision: from myriad concepts available, which relatively few are relevant to the current episode? Such choices, in addition to the consequences within a larger system (such as leading to actions affecting the external world), affects the generation of future concepts (Figure 3B). In this sense, interpretation is an active process.

We note that there are a number of alternatives in the design of both the interpretation data structures and algorithms. We describe a preliminary approach that has worked well but we expect much improvements and extensions are possible. For example, currently, in our implementation, only exact matching is allowed (see Section 3 for relaxations).

#### 2.2.2. Interpretation chains (data structures)

The interpretation data structure is primarily a chain of a sequence of concepts from the same level, one (concept) sequence for each level, the sequence at level 0 leading to a sequence at level 1, which leads to a sequence at level 2, etc. As an example, the sequence $\left[C_{1},C_{3},C_{1}\right]$ has three concepts: $C_{1}$ is at concept position 0, $C_{3}$ at position 1, and $C_{1}$ (again) is at position 2. For each position of a sequence, we keep an interpretation slot object that has a pointer to the concept used and contains other information, including match information (exact match to the lowest level). If the original input line is “book,” then the sequence at level 0 is [*con*_0_(“b”), *con*_0_(“o”), *con*_0_(“o”), *con*_0_(“k”)]. In our current implementation, all character positions (primitives at level 0) need to be covered by (matched against) exactly one concept at each of the higher levels (thus, leading to the requirement of exhaustive and non-overlapping coverage) and vice versa (non-redundancy): each (higher level) concept in the sequence has to cover (account for) at least one position in each of the lower levels. In Table 2, the first concept in layer 3 segmentation, *con*_3_(“reg”) covers *con*_2_(“re”) and *con*_2_(“g”) in layer 2, and ultimately covers the first three positions in layer 0. We note that a concept can occur multiple times in an interpretation.

In future, we want to relax both conditions of partitional and completeness, e.g., allow interpretations in which concepts can overlap to an extent in what they cover, as well as allow some mismatches or approximate matches to handle noisy input (Section 3.7).

#### 2.2.3. Interpretation: A search process

The process for finding a good interpretation is a beam search and proceeds one layer at a time. The interpretation sequence at layer *i* yields one or more candidate sequences at layer *i*+1, thus one or several (partial) data structures (chains of sequences) are created, each corresponding to a different search path. A search path is finished once a sequence at the highest level is created, and the data structure is complete. One such structure, among several candidates, is picked based on the average CORE score (Section 3) and used for updating (online learning).

The process of segmenting a layer *i* sequence to get one or more candidate interpretations at layer *i*+1 is the same for all layers *i*. Given a layer *i* sequence, initially, all its concepts (positions) are marked uncovered. We picked a remaining uncovered concept at random. The concept's holonyms, as well as its clone, are then matched against the sequence. The clone always matches, and a few holonyms may match too. One of the matches is picked by a certain quality score. We use the concept's historical CORE if it is at the top level (the current highest layer of the system), and otherwise, the historical average CORE of holonyms it leads to. The matched one or two concepts in layer *i* are marked covered, and we repeat the process for the remaining uncovered concepts in layer *i* until all are marked covered. Once all are covered, we have a candidate interpretation (sequence) at layer *i*+1. By default, we tried 10 times from each sequence and kept the best three sequences at the next level (the beam width parameters). For computing sequence scores (CORE scores), we need to compute prediction probabilities (described next).

#### 2.2.4. Deriving prediction probabilities

For a concept C at any position in an interpretation sequence, the probabilities from the context, concepts within Δ positions, are aggregated and normalized to obtain its prediction probability at that position, denoted pred(C).

For example, let us assume the context size is 1, and the system does bidirectional prediction (both sides of each position predict). For position 1 in the sequence $\left[C_{1},C_{3},C_{4}\right]$, concept $C_{1}$ is on the left of the position, and concept $C_{4}$ is on the right. Let us assume $C_{1}$ has two edges for its (relative) position +1, say to $C_{2}$ and to $C_{3}$ with weights (probabilities) 0.1 and 0.15, respectively (thus, $w_{C_{1},C_{3},1}=0.15$). Let us assume $C_{4}$ has one edge only to $C_{2}$ with weight 0.11 ($w_{C_{4},C_{2},-1}=0.11$). Thus, we say $C_{1}$ predicts $C_{2}$ and $C_{3}$ while $C_{4}$ predicts $C_{2}$ only. Before normalizing, $C_{2}$ and $C_{4}$ get raw prediction scores of 0.1+0.11 = 0.21 and 0.15, respectively, and after normalizing, we get $\text{pred}\left(C_{2}\right)=\frac{0.21}{0.36}$, and $\frac{0.15}{0.36}$ for $C_{3}$ (and all other concepts are implicitly at 0).

We have experimented with different normalization techniques, such as softmax, as well as using learning techniques to extract probabilities. Plain linear normalization, as explained above, does sufficiently well in our experiments.^7^

#### 2.2.5. Scoring the interpretations

Not all interpretations (concept sequences) are equal. For instance, a good interpretation of “anewbike” is the sequence “a,” “new,” and “bike,” assuming the system is exposed to much English text, and all three concepts have been learned sufficiently well. However, in this situation, if the system initially joins “a” and “n” together to get the concept “an” and commits to it,^8^ we get an inferior interpretation for the rest of the string “ewbike.” The system performs a beam search to pick the most promising interpretation at the highest level. In order to select a final (interpretation) chain, the highest level sequence is scored from each chain, which is simply the average score, given below for a sequence containing *k* concepts (CORE is described in the next section):


(1) $$\begin{array}{l} \text{InterpretationScore}\left(\left[C_{1},C_{2},\cdots \;,C_{k}\right]\right)=\frac{1}{k}\underset{1\leq i\leq k}{\sum }\text{CORE}\left(C_{i}\right) \end{array}$$

A new holonym C (at level 1 and higher) poses a special challenge, as it needs to be used, so other concepts can develop prediction weights to it, and pred(C) is sufficiently well estimated, before CORE(C) can be well estimated. This presents a chicken-and-egg problem (Madani, 2021a). Currently, our solution is to include an *exploratory* period. For a new holonym, the system assigns an optimistic probability of 1.0 in place of actual pred(C), so a new concept gets used when it matches the primitives.^9^ A counter is kept with each concept, and once the counter reaches a limit (50 in our experiments), the normal pred(C) is used.

For scoring lower sequences, we use the average of the historical CORE scores of concepts in the sequence. We note also that initially, when there are only primitives in the system before layer 1 is added, the interpretation task is trivial, as there are no higher level holonyms.

#### 2.2.6. Online updates of prediction edges

In each (online) episode, a final interpretation chain, out of several candidates, is selected based on Equation (1), and is used for updating edge weights and other statistics. For example, if the selected top-level sequence is $\left[C_{4},C_{3},C_{2},C_{5}\right]$ with Δ = 2 (and bidirectional), updates for position 0 involve strengthening the edge weights from $C_{3}$ and $C_{2}$ to $C_{4}$ ($w_{C_{3},C_{4},-1}$ and $w_{C_{2},C_{4},-2}$ are increased). Similarly, for position 1, $w_{C_{4},C_{3},1}$, $w_{C_{2},C_{3},-1}$, and $w_{C_{5},C_{3},-2}$ are increased. Edges to other concepts (if any) are weakened, as explained in more detail next.

The edge weights are updated using (sparse) exponentiated moving average (EMA) updates (Madani and Huang, 2008). EMA enjoys a number of useful properties, in particular, for handling non-stationarity in time series and finds applications in financial and economic modeling. Let *r* denote the learning rate, *r*∈[0, 1]. Sparse EMA updating consists of weakening the weight of all edges of a concept for a given position *via* multiplying by 1−*r*, and then adding *r* to the weight of edge connecting to the observed. Let us imagine $C_{2}$ appears right after $C_{1}$, i.e., $\left[\cdots \;,C_{1},C_{2},\cdots \;\right]$, and before update $w_{C_{1},C_{2},1}=0.4$. Then, after update, it becomes (1−*r*)0.4+*r* = 0.36+1 = 0.46. Let us assume, before update, *w*_*c*_1_, *c*_3_, 1_ = 0.5 ($C_{1}$ is also connected to $C_{3}$). After the update, it is weakened to $w_{C_{1},C_{3},1}=0.5\left(1-r\right)=0.45$. One can verify that the weights remain in [0, 1], and, for a specific position, sum to no more than 1.0 ($\forall C,i,w_{C,*,i}=\underset{j}{\sum }w_{C,C_{j},i}\leq 1$, or the weights of a position, at any time point, form a *sub-distribution*, with 1.0 being a fixed point for the total weight mass). Moreover, under fairly general assumptions (e.g., taking into account a possibly changing learning rate *r* of EMA and the budget on the number of edges), for each position, the weights converge to approximate conditional probabilities. For instance, the weight $w_{C_{1},C_{2},1}$ converges to the probability of observing $C_{2}$ immediately in the next position, given $C_{1}$ is observed in current position. We note that each node, for each position, keeps its own gradually decaying learning rate, and it is shown that the harmonic decay of rate (starting high and lowered with each subsequent episode, *via* a harmonic schedule) has benefits for faster convergence (Madani, 2021a).

#### 2.2.7. Other online updates

The active concepts update a number of other statistics and scalar fields, some of these are important for guiding the search during interpretation (Section 2.2.3). The concepts at the top level update their historical CORE values (*via* a moving average such as EMA). All other active concepts (at all levels other than the top) update their historical CORE based on the CORE of the concept at the top level they lead to.

### 2.3. Offline tasks

Some tasks may need to be performed every so often. They may also be more global (than the online tasks), i.e., may need to process a relatively large number of concepts and/or edges, so they may be expensive to run more frequently. However, if performed only periodically, for instance, once the cost is amortized over all (online) episodes, the extra cost becomes feasible. These offline tasks include organizational tasks, such as garbage collection (e.g., recycling concepts no longer used). Some learning tasks that require the statistics gathered over many online episodes and do not require any single specific episode, such as creating new concepts or simplifying and transforming existing ones, should be performed in the offline phase too. We explain two main offline tasks we have implemented.

#### 2.3.1. Composing new concepts

Putting together any pair of concepts to create new concepts would lead to too many new candidates. Even if two concepts are seen together, this occurrence could be spurious, and furthermore, we would still get too many such co-locations for subsequent analysis and use. In our current implementation, we consider a pair of concepts, $C_{1}$ and $C_{2}$, a good pair, for creating a holonym when we have strong evidence that $C_{2}$ follows $C_{1}$ with probability greater than the prior of $C_{2}$ (of observing $C_{2}$ in a top-level interpretation). When the test is satisfied, we have strong evidence that $C_{1}$ followed by $C_{2}$ can be part of a larger concept. We use the approximate binomial tail test of significance, which is based on KL divergence (Arratia and Gordon, 1989; Ash, 1990; Madani, 2021a,b). This test works better than the commonly used pointwise mutual information when $C_{2}$ is relatively frequent (Church and Hanks, 1990; Manning and Schutze, 1999).

#### 2.3.2. Adding a new layer

In the offline phase, we may also add a new layer occasionally. When adding a new layer, the system performs the following. All existing concepts in top layer *i*, each gets cloned for new layer *i*+1, each clone getting the prediction edges to other clones in layer *i*+1 (so, if $C_{1}$ has an edge to $C_{2}$ in level *j*, the clone of $C_{1}$ gets an edge to the clone of $C_{2}$ in level *j*+1), and historical scores (used for guiding interpretation) are copied appropriately. In addition, composition criteria are checked for creating new (non-clone) holonym concepts for the new layer *i*+1. All concepts at *i*+1 are appropriately initialized (frequencies initialized to 0, optimistic historical probabilities at 1 for non-clone concepts, and empty lists of prediction edges and part of edges).

### 2.4. Timings and computational costs

Each online phase analyzed 1,500 lines (episodes), which took 3 min each when layer 1 was the maximum layer, with the default of 10 tries and keeping 3 for each beam search level. It took 30 min when the maximum layer was level 4 (e.g., for Model4). We note that one could train models in parallel and periodically aggregate the models. Model sizes also grow with more episodes and layers (additional concepts and edges), from a few megabytes (MBs), compressed, when layer 1 is the maximum layer, to low 100s of MBs for Model4 in our current experiments. The main time complexity is in the search for a good interpretation, which primarily depends on the width of the beam search. Madani (2021a) discusses size budgets, e.g., on node outdegrees, that keep the computations efficient.

## 3. Scoring *via* CORE

We first develop the score for the case of string concepts with an exact match, which applies to the current implementation of Expedition. We, then, generalize the score to generative models (Section 3.6) and describe a related extension for inexact match. We conclude with an empirical comparison to n-gram and neural network language model techniques, on character prediction.

We want to encourage the system to learn larger patterns. Fundamentally, it is plausible that an organism that can predict larger patterns in a single prediction attempt, whether the pattern extends further into the future or farther spatially, has a survival advantage over those predictions that are more myopic. Furthermore, meaningful n-grams (e.g., words) are more powerful predictors than single characters: words predict other nearby words (longer than characters), while characters are best at predicting other nearby characters.^10^ A single character, such as “z,” has significant predictive power over a few character locations nearest to it, while the word “zoo” has significant predictive power over a few word locations (many more characters). Thus, beginning at a relatively low level such as characters in the text, it pays to acquire larger patterns, in terms of both predicting such patterns, and using them as predictors.

To assess the quality of a candidate interpretation, we imagine comparing the system against some reference or baseline system. In particular, here, we will use a very simple character-level predictor: the baseline never learns larger patterns. Let us say Expedition is assigning pred(C) (Section 2.2.4) to a string concept C that occurs in (covers part of) the input. The baseline makes the independent assumption when predicting and does not use any context. Therefore, it assigns ∏_*i*_prior(*c*_*i*_) to the concept C corresponding to *c*_1_*c*_2_⋯*c*_*k*_ (irrespective of the context in the interpretation).^11^ We define CORE to be the log of the ratio as follows:


$$\begin{array}{ll} \text{CORE}\left(C\right) & =\log\left(\frac{\text{pred}\left(C\right)}{\underset{1\leq i\leq k}{\prod }\text{prior}\left(c_{i}\right)}\right) \end{array}$$

(2) $$\begin{array}{l} \text{(concept}C\text{corresponds to}c_{1}\cdots c_{k}\text{)}. \end{array}$$

This is the reward of the system for predicting concept C with probability pred(C). The farther the system gets from the baseline in the above sense, i.e., the larger the ratio, the higher the system's CORE score.^12^ The priors of the primitives (their occurrence probability) can be updated in each offline phase, or in an online manner (e.g., *via* EMA) during processing each episode.

### 3.1. A related view: A combination of two types of fit

**Figure 5:**
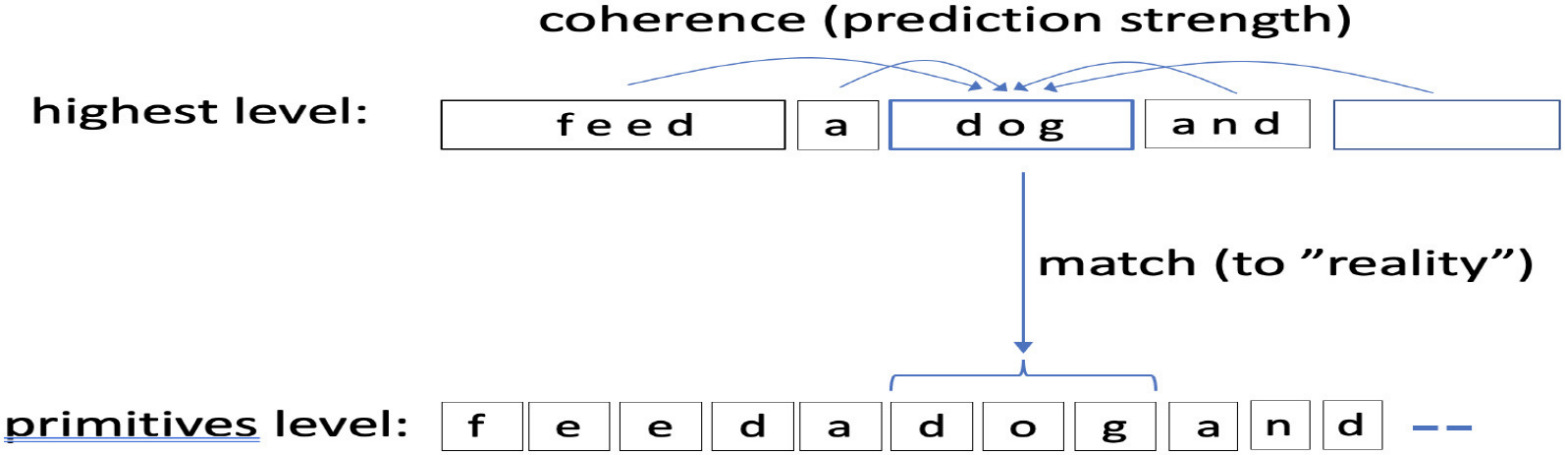
A picture of scoring *via* CORE. CORE is computed for each position in the highest level concept sequence. CORE combines two measures: a match score to lowest level primitives (reality match) and how well the concept fits with its surround, or coherence, for which we use pred(C) (the probability the concept attains from other predictors in its context). In this picture Δ = 2.

The CORE score can also be viewed as a tradeoff between two types of fit: (1) how a candidate concept “fits” with other concepts in a candidate interpretation (laterally), and our measure for this fitness is how well a concept is predicted, i.e., the probability that it attains from the local context (Section 2.2.4), and (2), and how well the concept matches (explains) the ground-level primitives it covers and the reward from that match. Figure 5 shows this setup.

Formally, we define the match reward of a concept C corresponding to *c*_1_*c*_2_⋯*c*_*k*_ as follows:


$$\begin{array}{ll} \text{MatchReward}\left(C\right) & =-\log\left(\underset{1\leq i\leq k}{\prod }\text{prior}\left(c_{i}\right)\right)=-\underset{1\leq i\leq k}{\sum }\log\left(\text{prior}\left(c_{i}\right)\right) \end{array}$$

(3) $$\begin{array}{l} \text{(}C\text{corresponds to}c_{1}\cdots c_{k}\text{)}. \end{array}$$

Thus, longer concepts (concepts with more primitives) and concepts with more infrequent primitives (pack more surprise) have a higher intrinsic reward. When interpreting, the intrinsic or matching reward of a concept C is balanced against how much probability the rest of the interpretation (the concepts in the context) assigns to C (the coherence part) to get a COherence + REality match, or CORE, as follows:


(4) $$\begin{array}{l} \text{CORE}\left(C\right)=\underset{coherence}{\underset{︸}{\log\left(\text{pred}\left(C\right)\right)}}+\underset{match\;\left(to\;reality\right)}{\underset{︸}{\text{MatchReward}\left(C\right)}} \end{array}$$

Notes: log(pred(C))≤0, so it is a negative (penalty) term in general. The CORE score for a concept and the average CORE for an interpretation can be negative, for instance if the prediction probabilities are not estimated well. In certain cases, it could be beneficial to weight the two coherence and match components of CORE differently. Finally, when different interpretations at the top level result in the same matching scores, ranking them based on average of CORE reduces to ranking based on prediction probabilities, such as the product of the received probabilities if context size is 1 and we always use one side (e.g., the left side) for prediction.^13^

### 3.2. When does it pay to compose?

One way to see how the above CORE objective promotes using composition (larger) concepts is to consider whether to join primitive $C_{1}$ (for a letter *t*_1_) with primitive $C_{2}$. We denote the holonym by $C_{1}C_{2}$ (more accurately, *con*_1_(*t*_1_*t*_2_)). A similar analysis holds for larger string concepts. Let $\text{prior}\left(C_{i}\right)$ denote the priors and let $P_{C_{i}}$ denote the historical average of the probability $C_{i}$ obtains, the average of $\text{pred}\left(C_{i}\right)$ over interpretations it occurs in. Let us assume the composition $C_{1}C_{2}$ would be predicted on average with probability $P_{C_{1}C_{2}}$. Now, it is often the case that $P_{C_{1}C_{2}}<\min\left(P_{C_{2}},P_{C_{1}}\right)$ (the system takes a hit in terms of raw probability, by combining). However, on average, the system predicting the holonym $C_{1}C_{2}$ does better in terms of interpretation average CORE (Equation 1) than the system predicting $C_{1}$ and $C_{2}$ separately if $\frac{1}{2}(\log(\frac{P_{{\text{c}}_{1}}}{\text{prior}({\text{c}}_{1})})+\log(\frac{P_{{\text{c}}_{2}}}{\text{prior}({\text{c}}_{2})})))<\log(\frac{P_{{\text{c}}_{1}{\text{c}}_{2}}}{\text{prior}({\text{c}}_{1})\text{prior}({\text{c}}_{2})})$. This is equivalent to ${\left(\text{prior}\left(C_{1}\right)\text{prior}\left(C_{2}\right)P_{C_{1}}P_{C_{2}}\right)}^{0.5}<P_{C_{1}C_{2}}$. In general, $P_{C_{i}}>\text{prior}\left(C_{i}\right)$ (the context helps in predicting) and replacing $\text{prior}\left(C_{1}\right)\text{prior}\left(C_{2}\right)$ by $P_{C_{1}}P_{C_{2}}$, we get that, often, as long as $P_{C_{1}C_{2}}>P_{C_{1}}P_{C_{2}}$ (more accurately, whenever $P_{C_{1}C_{2}}>\max\left(P_{C_{1}}P_{C_{2}},\text{prior}\left(C_{1}\right)\text{prior}\left(C_{2}\right)\right)$), we have improved the average CORE of the system by composing.

Of course, in general, the historical prediction probability of a composition is not available when composing: the system needs to join first and, at some point, may get sufficient evidence that, for instance, $p_{C_{1}C_{2}}>p_{C_{1}}p_{C_{2}}$. We want to create concepts for which an improvement in CORE is likely true (see Section 2.3.1). Furthermore, there can be multiple competing candidates, compositions (in an episode), and we may need additional criteria to prefer one over the others.

### 3.3. Connections to character-level entropy and KL divergence

**Figure 6:**
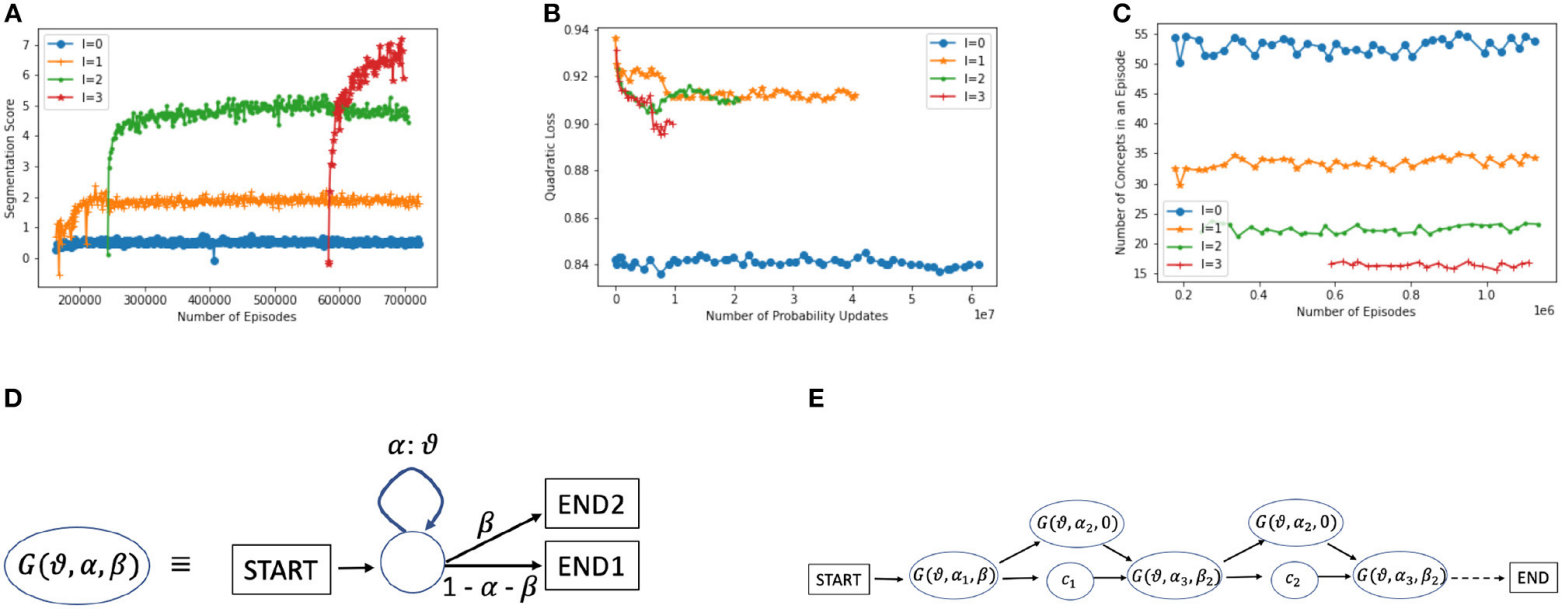
**(A)** Progress in interpretation scores (average CORE) at different levels (Equation 1). **(B)** Squared loss on predicted probabilities per level. **(C)** Number of concepts, on average, per episode at each level (≈55 primitives). **(D)** A universal generator G(V,α,β), to support approximate matching. With α, a (small) positive probability, G(V,α,β) can generate any string $T\in V^{*}$, with a positive probability. The two terminal states (when β>0) are for flexible transitioning see **(E)**. **(E)** During a match attempt, a few versions of the universal generator can be inserted into a string concept $C=c_{1}c_{2}\cdots c_{k}$, as shown. This allows for skipping, replacing, prepending, or appending to each letter of C.

If the system remained at the character level (did not generate new concepts), then the maximum CORE possible would be reached if it could predict each character with certainty (probability 1). The maximum achievable expected CORE, using Equation 2, would then be the character-level entropy of the input stream: $\underset{c\in V}{\sum }\text{prior}\left(c\right)\log\left(\text{prior}\left(c\right)\right)$ (where the sum goes over all the primitives or characters in the alphabet V). For instance, the character-level entropy is approximately 4.45 bits on NSF abstracts. This maximum score is very hard to achieve at the character level, and it is surpassed with higher levels, as shown in Figure 6A. At level 0, the system reaches an average CORE of 0.7, but at level 2, the CORE is approximately 5, and with level 3, it reaches approximately 8.

One can define the utility of a concept C as a predictor for a fixed position, let us say position 1, in terms of expected CORE. At the character level, this is roughly the KL divergence of C's edges for that position: $\underset{c_{i}}{\sum }w_{c,c_{i},1}\log\frac{w_{c,c_{i},1}}{\text{prior}\left(c_{i}\right)}$. Because *w*_*c*, *, 1_ ≤ 1, this is an approximation to KL. However, an easy adjustment is to assume the remaining mass is spread over remaining (0 weight) concepts, proportionate to their prior concepts, Then, prediction utility becomes KL divergence.

### 3.4. Uses of CORE

We use CORE during interpretation searches to find a good top-level interpretation. Each higher level concept keeps a track of its historical (average) CORE and this is used during bottom-up interpretation search (Section 2.2.3). We use the interpretation score (incorporating CORE, Equation 1) averaged over many episodes as a measure of progress of the system (a measure of prediction accuracy), as shown in Figure 6A.

In Table 3, a few concepts learned with a few statistics, such as the number of times observed (in their layer) and their historical CORE scores (a moving average over episodes they appear). Longer concepts (longer strings) tend to get higher scores. We note that “sand” appears to be mostly a concatenation of “s” and “and,” such as “ projects and.” and the proper word “sand” occurs far less than the frequency in the table indicates. This is an undesired use of “sand” (recall that the system does not see blank spaces separating words). With more training and inference, these errors go down. For instance, we have observed that the ratio of bad to good splits goes down with inference time (higher search width) and concept level, and it is positively correlated with CORE (Madani, 2021a).

**Table 3:** Statistics on a few concepts in Model3: Left columns show “ther” concepts (two different levels) and a few related ones (e.g., “whether”), and right columns show a few most frequent. The frequency (number of times seen), how many episodes ago it was last seen in an interpretation, from the time the snapshot was taken (e.g., *con*_2_(“ther”) was seen 22 episodes ago), and the historical CORE of the concepts are shown. The right column shows non-clone concepts with the highest observation frequency at level 3, except for the last row, *con*_3_(“s”), which is the concept with the highest frequency in level 3 (it is a clone).

**Concept**	**Frequency**	**Last seen**	**CORE**	**Concept**	**Frequency**	**Last seen**	**CORE**
*con*_2_(“ther”)	57,890	25	14.0	*con*_3_(“sand”)	54,456	24	10.1
*con*_3_(“ther”)	6,370	106	8.0	*con*_3_(“research”)	50,353	20	25.3
*con*_3_(“there”)	4,023	58	10.4	*con*_3_(“project”)	42,501	22	28.0
*con*_2_(“with”)	84,643	16	17.1	*con*_3_(“ation”)	36,479	101	13.4
*con*_3_(“with”)	22,195	48	10.8	*con*_3_(“develop”)	28,092	85	25.5
*con*_3_(“whether”)	3,383	388	21.0	*con*_3_(“s”)	966,729	2	1.1

### 3.5. Other measures

We developed CORE as a principled smooth measure to guide interpretation. Perplexity (or equivalently entropy) is widely used in language modeling (Jelinek et al., 1977; Cover and Thomas, 1991; Rosenfeld, 2000), but perplexity deteriorates, in general, with larger vocabularies and requires an extension to handle vocabularies where multiple patterns with a common prefix (e.g., “b,” “ba,” “bat,” and “bath”) can occupy the same location of the input. Probability loss measures such as quadratic loss are smooth too, but deteriorate, in general, with a growing vocabulary (the loss can go up with level Figure 6B), and similar to perplexity, do not reflect the benefits of learning larger patterns. We experimented with quadratic loss and saw it converge very quickly, while CORE kept improving (Madani, 2021a). Measures such as the number of concepts in an interpretation per episode, as shown in Figure 6C, are informative but not smooth and can also converge quickly.

### 3.6. Beyond strings: Generative models

Let us assume now that concept C corresponds to a more general probabilistic generative model. Let *T* = *t*_1_*t*_2_⋯*t*_*k*_ denote a span of text or a string of characters, where $t_{i}\in V$. Let us assume Expedition wants to interpret (map) the string *T* in the input line as concept C. We want to compute the CORE, which we now denote by CORE(C,T). Let $P_{C}\left(T\right)$ denote the probability that C generates *T*. When C corresponds to a string *s*, as mentioned earlier, $P_{C}\left(T\right)\in \left\{0,1\right\}$ (it is 1 if *T* is the string *s*). The match (quality) score MatchReward(C,T) is defined as the ratio of the probability of generating *T* to the baseline's probability for string *T*, and CORE is now defined with respect to the quality of the match as follows (instead of assuming a perfect match as earlier):


$$\begin{array}{ll} \text{MatchReward}\left(C,T\right) & =\log\left(\frac{P_{C}\left(T\right)}{\underset{1\leq i\leq k}{\prod }\text{prior}\left(t_{i}\right)}\right) \end{array}$$

(5) $$\begin{array}{l} \text{(where string}T=t_{1}\cdots t_{k}\text{)} \end{array}$$

(6) $$\begin{array}{ll} \text{CORE}\left(C,T\right) & =\underset{coherence}{\underset{︸}{\log\left(\text{pred}\left(C\right)\right)}}+\underset{match\;\left(to\;reality\right)}{\underset{︸}{\text{MatchReward}\left(C,T\right)}} \end{array}$$

For example, if C is the stochastic disjunction of the numeric digits, {“0”,“1”, ⋯ , “9”}, each with (uniform) probability 0.1 and each with prior 0.01, then MatchReward(C, “2”$)=\log\left(\frac{0.1}{0.01}\right)=\log\left(10\right)$. We note that previously, with plain string concepts and $P_{C}\left(T\right)\in \left\{0,1\right\}$, we would simply not consider any inexact matching (the search process discards such). How efficiently and accurately MatchReward(C,T) is computed depends on the efficiency of computing $P_{C}\left(T\right)$, which, in turn, depends on the general complexity of the structure of concept C. For instance, if concept C corresponds to a string concept or an augmented version of it (see next), (approximately) computing the match score is fast.

### 3.7. Approximate matching

Approximate matching is the ability to tolerate some amount of noise or corruption in the input, such as a concept corresponding to “apple” matching variations such as “aple” (a dropped letter) or “applax” (the swap of the ending “e” with “ax”). The confidence of such approximate matches will depend, in part, on the relative number of matching characters and mismatches, and in part on the context, as we explain. Approximate matching is essential for error correction in real-world applications.

We note that the extensions we present here are tools used during inference at scoring (matching) time, and the structure of a learned concept is not (permanently) changed. The structure is only augmented at matching time for a string concept, as shown in Figure 6E. We will focus on string concepts. We expect that similar augmentation is applicable to more general structures, such as AND-OR trees.

For approximate matching, it is useful to think in terms of probabilistic string generation, which also connects to the CORE scoring. Figure 6D shows a universal generator, G(V,α,β), i.e., a probabilistic finite state machine that can generate any string in $V^{*}$, with some positive probability, where $V^{*}$ is the Kleene closure.^14^ We use $G\left(V,\alpha_{i},\beta_{j}\right)$ to denote different variants of the universal generator in the picture (e.g., they can differ in the transition probability α). To tolerate different types of mismatches flexibly, for string concepts, the inference engine in effect inserts variants of *G*() between every two consecutive primitives, and one *G*() on a branch skipping the entire character, as shown in Figure 6E. The α_*i*_ may depend on the length of the string and could be learned or tuned over time, and it is possible that fixed values may suffice in many domains.

In general, α_*i*_ and β_*i*_ are tiny, and the augmented automation generates the intended string corresponding to the concept with the highest probability. However, it also gives reasonable probability to strings with small deviations from the intended string (such as a single correct character swapped with another). Thus, whether a deviation is selected also depends on how well the concept is predicted (its coherence) and how well it predicts others and the score of other alternative sequences (in particular, alternatives include breaking the concept into two or more subconcepts).

As a concrete but rough example, let us imagine that the string concept “apple” is being predicted and let us consider that it is being matched against “abple” vs. “apple” (a single mistake, a swap of “p” with “b,” in the second letter position). We will do a rough calculation of the extent the MatchReward() score goes down. For the augmented “apple” (the corresponding concept) to generate “b” instead of “p,” the alternative path to *G*() needs to be taken, with say α = 0.05 and assuming prior(*b*)≾0.04,^15^ thus instead of getting a reward of roughly $\log\frac{0.95}{0.04}≿\log\left(20\right)$, one gets a cost of $\log\frac{0.05\text{prior}\left(“b^{\prime \prime }\right)}{\text{prior}\left(“b^{\prime \prime }\right)}\approx -\log\left(20\right)$. So, a mismatch of one letter costs (reduces the reward by) more than one letter, and it may even lead to a net reduction in reward of two average letters. However, the string concept for “apple” may best fit the context compared with the alternatives (e.g., breaking into two concepts “ab” and “ple”), ultimately leading the system to interpret “abple” as “apple.”

We note that one could add this type of flexible inference only after a period of learning, but we expect that it would be best to have approximate matching work together with the learning of concepts. We also note that finding the best alignment during a (string) match can be a costly task (e.g., involving dynamic programming), and an any-time search strategy that would quickly stop a match for pairs of strings with a highly likely large edit distance is preferred, especially when there already exist good alternatives (discovered by different search paths).

### 3.8. A comparison to language models

We have run a variety of experiments, as we develop the algorithms (Madani, 2021a). In particular, in one experiment, we converted the input text lines into binary sequences, i.e., the lowest level having only two primitives. The system was eventually able to recover higher level patterns. To situate Expedition with respect to current methods, we conclude the section with a comparison to two existing methods for statistical language modeling (SLM): neural network transformers (ANNs) (Vaswani et al., 2017) and n-grams (NGR) (Manning and Schutze, 1999; Rosenfeld, 2000). Transformers are the state of the art, and the progress in language models based on neural networks has substantially expanded the diversity of the applications of SLM (Dong et al., 2019; Brown et al., 2020; Rogers et al., 2020). Our techniques are close to NGR methods, in that they both use predictions of character strings (further discussed below). We note that we view this research as early stage, and both the problem formulations and algorithm development require further investigation. However, empirical comparisons such as those below help give an idea of where we stand in terms of performance on traditional tasks, as well as help to shed further light on the differences and similarities among the techniques. We, next, describe the prediction tasks and the evaluation criterion, and how each method is set up for the task (parameter settings, etc), then discuss the findings.

#### 3.8.1. The character prediction task

With our focus on starting concept learning at the level of characters in this study, we compare the task of character prediction. Unlike Expedition, neither (standard) ANN techniques nor NGR “graduate” from predicting single characters, or in general, they do not go beyond the vocabulary they start with, though both techniques are powerful and can be extended to predict further into the future by, in effect, simulation. In every test episode, each model outputs a probability distribution, containing |V| probabilities (summing to 1) (recall |V|=94). We use log-loss (or cross-entropy) for evaluation: $\frac{1}{N}\sum {\text{pred}}_{M}\left(c\right)$, where pred_*M*_(*c*) is the probability assigned to the observed (target) character *c* (hidden and to be predicted) by model *M* (in nats, i.e., using the natural log). This loss allows us to assess how much a trained model, with the help of a context, can reduce the raw (unconditioned) entropy (three nats), on average (Table 4). ANNs are also trained on the same loss (Section 3.8.4). The character string occurring before (to the left of) the character to be predicted is the context in these experiments and is the input to the model (an example is given below). Different techniques process this input differently. In all cases, blank spaces are removed, but before removal, they are used to determine which letter to predict, as described next.

**Table 4:** Log-loss performances for predicting the next character (the lower, the better, Sections 3.8.1, 3.8.2). NGR refers to using n-grams, losses shown for n-gram, length limits of 1, 2 (bigrams), and no limit (Section 3.8.5), ANN is a neural network (Section 3.8.4) and the setup for Expedition is described in Section 3.8.6. All losses except for a few improve as we go from 500 to 20k data, and the 1st-letter (“1st”) task, especially on (out-of-domain) NEWS, is the most difficult.

**Tasks (on 200 lines) → **	**1st**	**Last**	**Rand**	**1st**	**Last**
**Methods** ↓	**NSF**			**NEWS**	
**Train on 500 lines (500 data)**					
Unigrams only	3.84	2.2	2.7	4.1	2.85
Up to bigrams	3.82	1.7	2.5	4.3	2.75
NGR (all n-grams)	3.90	1.4	2.4	4.4	2.7
ANN (Transformer)	3.76	2.1	2.6	4.0	2.6
Expedition, 2 rounds	3.79	2.4	2.7	4.1	2.9
Expedition, 3 rounds	3.89	2.6	2.8	4.2	3.0
**Train on** ≈**21k lines (20k data)**					
Unigrams only	3.72	2.2	2.7	3.8	2.6
Up to bigrams	3.49	1.6	2.4	3.9	2.45
NGR (all n-grams)	3.47	0.68	1.8	4.3	2.3
ANN (Transformer)	3.22	0.76	1.6	4.1	2.1
Expedition, 2 rounds	3.43	2.1	2.5	3.9	2.7
Expedition, 3 rounds	3.52	2.1	2.6	3.95	2.7

#### 3.8.2. Three evaluation variations and an example

Each test line (not seen during training) yields one test episode. We report test performance on three settings. In the *1st-letter* setting (column “1st” in Table 4), the task is to predict the first letter of a word, where the context, fed to a model, is the string characters from the words before. In the *last-letter* setting, the task is to predict the last letter of a word, the context being the remainder of the word and all previous words. Finally, the *rand* task refers to picking a position uniformly at random (close to the middle of the line). We used the middle so that a sufficiently long context would be available. For example, for the line, “The proposed project ends shortly.,” the word “project” is in the middle of the line. The first-letter task is predicting the first letter “p” (of “project”), given the (left/preceding) context “Theproposed” (which is fed to the model). We note that a model may only use a portion of the given context. For instance, a 1-gram model (limited to using only unigrams) will only use the preceding letter “d” to predict the next letter (the rest of the context is ignored). If the model assigns say 0.2 to the correct letter “p” (pred_*M*_(“p”) = 0.2), its loss in this episode is −log(0.2). The last-letter task is predicting “d” given the context “Thepropose” (thus, this task is a pure word- or pattern-completion task). For all cases, for each test line, the middle of it is first picked, and either a random offset (within ±5 positions) from that point is picked to determine the testing (rand) or the closest left blank space is located, and an appropriate first-letter or last-letter task is generated. All models are tested on identical episodes. As explained below, with our current implementation of how interpretation is done, the first-letter task may be more appropriate for Expedition. The distinctions among tasks also shed light on the relative strengths of different methods.

#### 3.8.3. The data splits

Approximately 21,000 text lines (of NSF abstracts), randomly picked, are used for training, and another approximately 2,700 lines are used for each validation and test split. In addition, for a brief exploration of how much of what has learned transfers to a different English language genre, we also evaluate the learned models on lines from the newsgroups data (NEWS). These include discussion postings under diverse topics, such as religion, science, politics, and computers (Lang, 1995).^16^ All models are trained and tested on the same splits of data: 200 test lines (from each of NSF and NEWS) are used for the losses in Table 4. To get an idea of the learning curve (trajectory), we trained on 500 lines (500 data), 5,000 lines, and the full training set (roughly 21,000) or “20k data,” and we report on the 500 and 20k data (similar findings on 5,000). Variances, due to randomization inside an algorithm (such as during search for interpretation), are not reported in the table to avoid clutter, as they are relatively small and do not affect our findings. We discuss them briefly when the algorithms are presented.

#### 3.8.4. The ANN method

We use the PyTorch implementation of transformers for language modeling (Paszke et al., 2019), using log-loss for training. Every character position of every line in the training data, except for the first character, becomes a training instance, thus different training instances have different context sizes, up to a maximum. We experimented with the following parameters: the (maximum) context size (best was in 10s and we used 30), the number of heads, the number of layers, the embedding and hidden layer dimensions, and the learning rate and its decay schedule. We used log-loss on the validation split (equivalent to the rand task) to pick the best parameter setting. The performance change appeared smooth for the most part, especially as the parameters are increased (e.g., 50 dimensions vs. 100, or 10 heads vs. 15 heads). On the 20k training data, with 10 to 20 layers and 10 to 20 heads, the log-loss was brought down to just under 1.5 on validation (requiring nearly 100 epochs). The model with 20 heads and 200 hidden nodes (5 million parameters) reached a log-loss of 1.468 in 90 epochs (its log-losses are shown in Table 4), while a model with the same structure but with 50 hidden dimensions reaches just above 1.5. The log-loss results were similar among these networks. A network with 1 layer (200 dim) and 1 head reaches log-loss of approximately 2. On the 500 training data, smaller networks performed better, as would be expected: performance of a 2-layer 1-head network is shown, which reached 2.49 log-loss on validation. Training and inference are a function of model size, in addition to the training size, and ranged from minutes (for 80 or 100 epochs) to hours. Thus, the total exploration of parameters took several days. We note that character-based models may not in general work as well as their word-level counter parts in terms of prediction accuracy, but in some domains the equivalent of words may not be available, and there is work on narrowing the gap (Al-Rfou et al., 2019).

#### 3.8.5. The NGR (n-grams) method

We implemented our own character-based n-grams (NGR) technique, collecting all n-grams appearing with minimum frequency of 5, and computing the distribution of the next character (the *next-letter* distribution) for each. For instance, if the n-gram “proposed” appears before “project” 90% of the time in the corpus and the remaining time appears before “research,” then its next letter distribution has two entries (or edges): it contains “p” with 0.9 and “r” with 0.1. We note that the number of distribution entries (edges) for an n-gram can be at most |V| (94 in our experiments). Longer n-grams can be more precise than the shorter counterparts but can have a higher variance over what they predict due to lower frequency and thus less reliable probabilities, and many legitimate items (letters) may get 0 probability. In particular, log-loss does not work with 0 probabilities (infinite loss), and a test or prediction time, we smooth the distributions (also referred to as the backoff technique), which improves log-loss performance. We do this by mixing *via* a convex combination of the (next-letter) distribution of the longest n-gram that matches a context with the distribution derived (recursively) from all the shorter matching n-grams. Thus, if the context is “projec” and the n-gram “projec” (a 6-gram) is available (it passed the frequency threshold), then the next-letter distribution computed for “projec” is mixed with the distribution derived from recursively mixing all its shorter n-grams, i.e., “rojec,” “ojec,” all the way down to the 1-gram ‘c' (which also do pass the frequency threshold). The weighting in the convex combination is a function of the frequency of the longer prefix: if below cnt1, its weight is *w*_1_ = 0.05, and the distribution of the shorter prefixes gets 1−*w*_1_ (or 0.95), while above cnt2, *w*_1_ is set to 0.95, and *w*_1_ is linearly interpolated for in-between frequencies. We experimented with a few variations and set cnt1 = 5 and cnt2 = 50. Finally, we further smooth *via* mixing with the uniform distribution over the vocabulary with a weighting of 0.01 (weight chosen by a bit of experimentation) for the uniform prior (and 0.99 for n-grams' predicted distribution). The same final smoothing is also performed for the output of Expedition.

We made a few optimizations for faster training and testing, and training on the 20k data takes approximately 5 min. The number of n-grams generated and used rapidly grows with training size, as observed in Table 5. Table 4 shows the performance of NGR when n-grams are limited to unigrams (only use the single preceding character as predictor), up to 2-grams and with no limit, i.e., use all that match.

**Table 5:** Number of n-grams used by NGR and Expedition (Expd), in three rounds on 500 and 20k data. Expedition generates and uses far fewer concepts. The number of prediction edges of NGR (trained on 20k) is ≈ 1 million (or 5 edges per n-gram on average), while for Expedition (bidirectional, Δ = 1) it is ≈200k.

**n-gram length → **	**2**	**3**	**4**	**All**
NGR 500	439	1.3k	1.2k	5.4k
NGR 20k	2.3k	9k	24k	264k
Expd 500	375	381	77	927
Expd 20k	433	266	405	1,198

#### 3.8.6. Expedition (modifications and parameters)

Concepts (predictors) in Expedition predict strings of different lengths. To compare prediction performance at the character level, we added up the probabilities assigned to the first character of the predictions. Thus, for example, if “a,” “apple,” and “banana” are predicted, with probabilities of 0.2, 0.35, and 0.45, respectively, then the letter “a” gets a probability of 0.55 and “b” gets a probability of 0.45. We used Δ = 1 (bidirectional) in these experiments.^17^ Once the context is interpreted and a final top-level concept sequence is determined, the last (right-most) concept is used to predict the hidden character to the right. Its predictions are mixed with a uniform prior (0.01 weight for prior) for NGR, as explained earlier.

We organized Expedition training into rounds, each round involving three passes over the training data. In the first round, unigrams (primitives) are available, prediction weights among them are learned in the first pass, and compositions (bigrams) are generated, and statistics for these new concepts are learned in the second and third passes within the round, and a final set of bigrams is selected but used for performance evaluation in the next round only. Thus, the performance of Expedition in first round is that of 1 g NGR, as shown in Table 4. In the second round, final prediction weights among the selected bigrams and existing primitives (unigrams) are learned, and the performance is reported. Then, in a second pass, new compositions (up to 4-grams) are generated to be selected, and then used in the next (third) round. Concepts need to be matched and seen sufficiently often, to develop a sufficiently reliable distribution for use in interpretation, or to be fully incorporated, and therefore, the number of used concepts is far below what NGR generates and uses, as presented in Table 5.

The top 4-grams used, the highest by frequency (number of times used in final selected interpretations) are “tion” (1,085 frequnecy), “will” (459), and “ment” (417). Similarly, most frequent 3-grams learned are “and” (1,913), “pro” (1,546), and “ate” (1,430), and the three most frequent bigrams are “ti” (22,191), “he” (22,140), and “th” (19,574) (generated in round 1 and used in round 2). Timing: A pass over 20k lines with a totality of a few thousand concepts, each line requiring an interpretation with a beam width of 5, takes several hours, and the whole training of more than three rounds took 1.5 days. The final set of concepts was ≈1k (Table 5), with ≈4k part-related (vertical) edges and ≈100k prediction edges in each direction.^18^ In these experiments, after the third round, on average, a training text line led to ≈30 top-level concepts. Thus, on average, active concepts are just below two characters long (as lines are 55 characters long).

COherence + REality match CORE improves on training and test splits (Table 6), although after a few rounds, possibly due to overfitting, it starts to degrade somewhat on test splits (not shown). The overfitting occurs later on larger training sets. Expedition does not optimize log-loss directly, but log-loss shows a similar pattern to CORE, though it tends to degrade (on the test lines) sooner (in earlier rounds). Interpretation is a randomized search, and we used five tries (beam width) for each (test) line. The standard deviation (std) over log-loss (averaged over 200 lines) was low, for example, std=0.02 for Expedition, trained on 20k, on the first-letter task, with 10 trials (3.89 ± 0.02 in Table 4, and lowering beam-width of interpretation to 1, increases the std to 0.025). The model learned by Expedition performed similarly under many different runs on 500 data and a few runs on 20k (low variance).

**Table 6:** Expedition trained on NSF, always improves CORE on NSF tests, with more rounds or more data. CORE improves on NEWS test with more rounds too, but only on 20k data.

	**NSF**	**NEWS**
500, round 1	0.93	0.78
500, round 2	1.56	0.70
500, round 3	1.97	0.77
20k, round 1	1.03	0.87
20k, round 2	2.99	1.66
20k, round 3	3.37	1.50

#### 3.8.7. Findings and discussion

Table 4 shows the log-loss results. The character entropies of both the NSF and NEWS texts are ~ 3 nats.^19^ All methods perform better with more training instances (20k vs. 500), and for almost all tasks, the loss is well below the (raw or unconditioned) entropy of 3 nats, with the exception of first-letter tasks. NGR is a simple method that performs very well, especially in the last-letter task (or the word/pattern completion). The rand task is basically what NGR and ANN are trained for, and its difficulty (as seen by the loss numbers) lies somewhere in between the first-letter and last-letter tasks. The Expedition is competitive on the first-letter task, on both test sources, but has room to progress for the other tasks. With our constraints on concept generation and usage (i.e., final incorporation into interpretation requires being matched at least 60 times), only a few 1,000 concepts are generated on the 20k data and close to 1,000 are used, in contrast to NGR (Table 5). For NGR, one can raise the threshold on the required n-gram frequency, but at the expense of prediction performance, in general, all prefixes of a word need to be kept (much overlap and unnecessary redundancy). Expedition finds substantially fewer patterns but patterns that likely better generalize. A (near) perfect Expedition system would discover the words and phrases, and prediction relations among them, with superior space efficiency compared with NGR (and with competitive first- and last-letter performances). However, Expedition transfers the space savings to the time cost of interpretation. While we seek to advance the capabilities in interpretation (e.g., as further discussed next), the efficiency of interpretation needs to be taken into account, and there will always be some overhead associated with it.

Our current interpretation method can do a better job of the possibility that an acquired concept (a pattern) is only revealed partially.^20^ Even though such a concept only matches partially what is revealed, it can be the best option considering the context. We leave the investigation of how to make such decisions during interpretation to future work. More generally, smart segmentation and interpretation can allow for more flexibility and adaptation at model deployment time (changing future environments). For instance, let us assume that there is additional noise (than present during training) at model execution time. For example, a simple type of corruption is when the letters in the context are replaced with other randomly selected letters at a certain (noise) rate. We measured how the log-loss of ANN and NGR suffers in such a testing setting, and both rapidly degrade, as we corrupted the test contexts.^21^ Expedition acquiring larger patterns, and as Section 3.7 outlined, with approximate matching (at interpretation time) could better tolerate some amount of noise.

The NGR performs very close to ANN. The size of the training data is relatively small, and most of the regularity is captured *via* concatenation at this level. Thus, we expect the gap to grow with more data. We expect that we need to extend Expedition to go beyond concatenation in capturing more diverse regularities.

## 4. Related work

Our work builds on large-scale online *supervised* learning, especially when the number of features is large and problem instances are sparse (Rosenblatt, 1958; Littlestone, 1988; Yang et al., 1999; Hoi et al., 2018). We investigated efficient online learning under a large, possibly growing, set of classes (concepts), *via* (sparse, associative) *index learning* (Madani and Huang, 2008; Madani et al., 2009). For example, in some applications, the set of predictors (features) could range in the millions, the set of concepts in the hundreds of thousands, while in an episode (e.g., an instance to classify) 10 s to 100 s of features would be active. In our view, this is a fundamental problem for an intelligent agent with many concepts: which, relatively a few, of myriad concepts are active or relevant, given an episode (a sentence, a visual scene, etc.)^22^ (see also Goode et al., 2020 on the index analogy and engrams). A natural followup question, considering human intelligence, is how one can acquire so many concepts in the first place, i.e., whether one could implement a system that could *build or discover its own many concepts* over time, without external or manual supervision. The goal of predicting one's input and getting better with time appeared powerful and promising, and prediction is fundamental to brain functioning (Ballard, 2000; Hawkins and Blakeslee, 2004; Bubić et al., 2010; Siman-Tov et al., 2019; Gatti et al., 2021). We began investigating prediction games, a fill-in-the-blank or a sequence prediction task, where concepts would serve both as predictors as well as targets of prediction (Madani, 2007). The system could use any efficient technique (clustering, concatenating, and so on) in an online manner to build and use concepts and validate and shape them for purposes of better prediction. The present study in particular was motivated by the problem of robustly figuring out which concepts are active in an episode, taking into account how they fit or constrain one another in addition to how well they match the lowest level (matching alone is insufficient) and led to investigating interpretation methods with appropriate (unsupervised) objectives. Our study also took inspiration from the neuroidal model of the neocortex, and in particular *random access* tasks (Valiant, 1994). These tasks involve associating pairs or multiplicities of arbitrary concepts (learning to associate stored items) from a large space of acquired concepts, where network nodes are more programmable than commonly studied neural network models (Hetz et al., 1991; Marcus, 2001) (but see also Natschl'´ager and Maass, 2002). Interpretation is a central component of the semiotics approach to cognition and meaning making (Konderak, 2018; Kull, 2018; Raczaszek-Leonardi and Deacon, 2018), and our work also aligns with the constructivism theory of epistemology and learning (Fosnot, 2005). From a biological viewpoint, how high may the inference in our approach go? There is evidence that inference from higher level cognition does not penetrate the inside of the so-called early vision system, while early vision itself may be complex and support its own top-down inference with some problem-solving capabilities, providing structured representations (of objects) to its consumers in other parts of the brain (Pylyshyn, 1999).

The working of the Expedition system is closely related to language modeling *via* n-gram methods (Manning and Schutze, 1999; Rosenfeld, 2000), but the vocabulary there is fixed and given. Learning structure and in particular finite state machines can be prohibitive computationally and in terms of sample complexity, but there is progress and positive results empirically and in special cases (Ron et al., 1998; Verwer et al., 2013; Castro and Gavaldà, 2016), though the focus has traditionally been on learning a single machine vs. many. Vector symbolic architectures, or hyperdimensional computing, also begin with a finite alphabet (Gayler, 2004; Kanerva, 2009; Kleyko et al., 2021), but we are not aware of work attempting to expand such vocabulary in an unsupervised manner. Much work in computer vision also attempts to build and grow compositional hierarchies of visual features with a mix of supervised and unsupervised techniques and objectives (Bienenstock et al., 1996; Fidler and Leonardis, 2007; Zhu et al., 2008; Si and Zhu, 2013), inspired by earlier work, e.g., Geman (1999) and Biederman (1987), and inspired in part by findings about hierarchies in primate vision (Krüger et al., 2013). We hope to advance and complement this line of work with our emphasis on prediction and coherence as a driver for learning.

Artificial neural networks (ANNs) are universal function approximators (Hornik et al., 1989), and with advances of the past few decades (diverse architectures, development and advancement of backpropagation), ample data and computation have become highly powerful for extracting diverse regularities. Following the success of ANNs in (supervised) vision and speech domains (Hinton et al., 2012; Krizhevsky et al., 2012; LeCun et al., 2015), large language models *via* deep ANNs, using a number of techniques such as embeddings, prediction, and attention, have had substantial recent successes in various diverse NLP and related problems (Collobert et al., 2011; Vaswani et al., 2017; Dong et al., 2019; Brown et al., 2020; Rogers et al., 2020). In much of current study on the text, the networks begin with an existing vocabulary and the embeddings of that vocabulary as input, and it is remarkable that much powerful learning is achieved without the need of the complexity of segmenting. The regularities and constraints in the input become highly distributed in the connection patterns of the network, providing advantages not only in making connections among similar patterns but also potentially losing some structure (leading to slow learning, see below) and interpretability. A sparse mixture of experts (MOEs), that attempt to activate a small portion of the ANN on a per-example basis (conditional learning or gating), trained *via* backpropagation, have had success in further scaling and speeding up of ANN training and inference (Fedus et al., 2021). Our approach could be viewed as a discrete (and a more structured) solution to large-scale unsupervised learning.

Concepts are, on one hand, foundational to human cognition (Murphy, 2002; Rakison and Oakes, 2003; Cohen and Lefebvre, 2017) and are, on the other hand “maddeningly complex” (Murphy, 2002). Concepts are interrelated in diverse ways (part-whole, taxonomic, spatiotemporal, domain-specific, and so on), or put another way, concepts seem to enjoy rich “content” (or attributes, in terms of other concepts). The nature of concepts and how they are acquired and adapted over time, along with their rich relations and flexible use, remain largely a mystery. Considering the importance and utility of concepts for solving advanced information processing tasks, or symbolic computation under uncertainty, and the complexity of conceptual phenomena, a diversity of algorithms or (sub)systems, working together, is likely required (Marcus et al., 2014). It is a major open question whether existing ANN techniques, based on backpropagation which have now substantially advanced many machine learning applications, can be extended (e.g., perhaps in a *post hoc* manner) to support concepts or provide a basis for reaching the flexibility of human-level cognition. Symbolic and relation learning and symbol manipulation tasks, such as variable binding, are described and reviewed by Marcus (2001), and limits of backpropagation of ANNs (and several other ANN approaches), in this regard, are discussed (see also Marcus, 2018). The brain performs extensive internal communications, possibly *via* a symbolic language, among its parts (not just externally), and based on the analyses of the reliability (to noise) properties of discrete vs. continuous representations for the purposes of communications within the brain, Tee and Taylor (2020) conclude that the basic information representation is likely discrete. The extensive research study on the interpretability of the models learned (Carvalho et al., 2019) and the related issues of model robustness and brittleness (adversarial attacks) (Szegedy et al., 2014; Ilyas et al., 2019) may also be linked to the major question of whether (backpropagation) ANNs can efficiently learn explicit discernible concepts with some robust internal structure.

## 5. Conclusion

We presented a system, composed of multiple learning and inference components, that over time learns to better interpret and predict its text world, by acquiring larger string concepts. The networked vocabulary of concepts that it maintains and grows includes a part-whole hierarchy and an association network. An unsupervised information-theoretic objective drives the learning of new concepts and their use when interpreting. We conjecture that learning concepts, or explicit structures, offer a number of advantages, such as learning (meta) patterns over concept structures downstream, for interpretability and communication, and for easier adapting to changes when deployed (robustness). More generally, we hypothesize that acquiring such vocabularies is an essential step for symbolic computations.

We highlight and summarize a few aspects of the approach that are differentiating, from the main current work on neural networks, and also ask whether the functions are biologically plausible.

- **Open Ended, Sparse Network:** The learning is open-ended in that the sparse network grows over time without a priori bounds, as a function of training data: edges and nodes are added (and discarded) as needed.^23^ While the brain is a physically bounded structure, it may be useful to model some of the learning processes as online tasks without a priori bounds on the structure.
- **Sophisticated Book Keepers:** Nodes, and to a lesser extent edges, do significant processing, e.g., to make connections and update weights (conditional probabilities), and they keep rich state in terms of several variables, such as their own learning rates and historical (prediction-related) rewards.
- **Complex Costly Interpretation:** The approach presented requires significant complexity in data structures, especially during interpretation, such as keeping and updating accurate pointers to active (matching) concepts and the portions of the input buffer they cover, and the associated serial processing necessary (code/engineering complexity).
- **Systems Learning:** From the outset, the idea of a system that used multiple interacting components, such as one for building concepts and another for learning to use them (e.g., for prediction), appeared promising, even at an early stage and for the relatively lower level (but complex) pattern recognition tasks. There is much evidence that learning, even for the same general goal, is achieved *via* multiple (cooperating and competing) processes in the brain (Ashby et al., 1998; Poldrack and Packard, 2003; Ashby and Valentin, 2017). Investigating the interactions in terms of game theoretic ideas should be fruitful.

We hope that this research can complement findings in neuroscience and cognitive psychology, in particular in the area of perceptual learning and development.

We plan to explore and advance interpretation further (such as exploring approximate matching). We are also investigating concept generation and assimilation, and in particular algorithms that learn patterns with more elaborate internal structure, e.g. containing variants of disjunctions. The external world enjoys a hierarchical structure (Simon, 1996; Callebaut and Rasskin-Gutman, 2005), and that aspect may allow for a more powerful learning that also benefits from a hierarchical as well as a modular nature. The current n-gram learning is a special case of learning pure conjunctions (spatiotemporal conjuncts). We hypothesize that supporting learning disjunctions in concept structure, such as discovering the numeric digits as a concept at some time point ({0, 1, 2, ⋯ , 9}) and building upon such to acquire patterns such as calendar dates and phone numbers, is feasible and would be powerful.

Future directions also include extending the approach to other modalities, such as sounds and images. Keeping the code complexity, the complexity of the algorithms, in check, in addition to efficiency, will remain a challenge, as we strive to extend functionality. The various dimensions of control, such as making the choice of what to input or to attend to, and to learn from, in a rich environment, as well as how to act externally (using what has been learned) are all important and fundamental directions. We hope to contribute to an understanding of how different processes, of learning and inference, could interact with one another, over the short and long term, and lead to robust development, through advancing the prediction games approach.

## Data availability statement

Publicly available datasets were analyzed in this study. This data can be found here: https://archive.ics.uci.edu/ml/index.php.

## Author contributions

The author confirms being the sole contributor of this work and has approved it for publication.

## Footnotes

^1^The learned patterns are at a relatively low level, and instead, we could refer to such as proto-concepts, or percepts, or (recurring) patterns, etc. For simplicity, and for our ultimate aim of shedding light on the origins of higher level concepts, and because these patterns are learned (e.g. as targets of prediction), we refer to them simply as concepts.

^2^A variety of discretization techniques exist for continuous data, such as clustering and vector quantization.

^3^One could also start at the word level. We start with characters to more easily test whether the errors accumulate and propagate on the path to reaching the relatively distant and the higher level of words and phrases (as the system builds upon its earlier constructions): good systems and algorithms should be robust against mistakes and some level of noise. Note that some human languages do not have natural word separators, and in some domains such as for DNA sequences or image processing, one may have to begin at a relatively low level (the equivalent of words and phrases may not be available). We have also explored converting English text to binary, i.e., starting at the level of only 2 primitives, and learning to reach the character level and beyond from such binary sequences, with promising results (Madani, 2021a).

^4^More generally, one could also keep edges that are position or direction insensitive, but we have not experimented with such (direction is highly informative for natural language text).

^5^The system could keep the number of edges under a budget for (prediction) efficiency.

^6^In general, segmentation may also include discarding parts of the input of the episode or requesting additional lines to be input to better determine the beginning and ends of concepts and related groups of concepts (such as determining clause or sentence boundaries). We expect differentiating between the two tasks will be fruitful in the future.

^7^Learning a mapping from raw scores and concept ranks to probabilities improves the quality of the probabilities in terms of quadratic loss. However, additional experiments may be required to determine whether any improvements attained from the trajectory of learning is worth the extra overhead.

^8^The question of commitment depends on the details of the interpretation algorithm. Our search technique for interpretation do not undo earlier decisions in a search path, but do explore several paths using beam search.

^9^If the length of the exploratory period is long enough, this can lead to a possible over use and an over-estimate of pred(C). Note also that this solution makes the implicit assumption that a sufficient portion of a time, when a concept matches the primitives, it is the right concept. Because, the system has somewhat strict criteria in creating new holonyms, this assumption is overall useful.

^10^Beyond language and text, our world is made up of complex systems that interact, and it has been observed that complex systems, the patterns of interactions, which can be used for predictions, tend to be hierarchical, i.e., components interact directly with other components that are mostly at the same rough level (cross-level or “vertical” interactions being limited to a component's own subparts and containers) (Simon, 1996; Callebaut and Rasskin-Gutman, 2005).

^11^Other baselines or reference systems (weaker or stronger) are possible too. In particular, here we are implicitly giving the baseline the length of the string it should generate. See the universal generators in Section 3.7.

^12^Anticipating that a sophisticated system will beat the simple baseline by a substantial amount, and so the ratio can be large, we take the logarithm.

^13^In this respect, while the probability assigned to an individual top-level concept goes down as concepts grow, the combined (product) probability assigned to longer stretches of text (words, lines, passages) can go up as higher level concepts are acquired and used (see Section 3.2).

^14^If V={“a”, “b”} (an alphabet of size two), then $V^{*}$ is the infinite set of all strings on *a* and *b*, i.e., {λ, “a,” “b,” “aa,” “ab,” “ba,” “bb,” “aaa,” ⋯ } (where λ denotes the empty string).

^15^Just under 100 unique characters, in our experiments, thus a uniform prior of 0.01.

^16^We did minimal preprocessing: we kept lines that began with a lower-case letter and that were at least 10 tokens (space-separated words) long.

^17^Larger window size can improve prediction performance, but can require more weight tuning. For speed of training and simplified comparisons, we set Δ = 1.

^18^Both directions are used in the process of generating the best interpretation, but one direction is needed for assessing log-loss performance.

^19^Equivalent to a confusion among roughly exp(3) or ≈20 equally likely possibilities, on average (while |V|=94).

^20^In our current set up, during training after a final interpretation data structure is selected, in part for efficiency, only concepts at the top level update prediction edges to one another, and the lower level concepts only update their scores so that the search to top level interpretation would be more successful in future interpretations. Thus the prediction weights of lower level concepts can become stale. During testing, our current interpretation method only interprets into whole concepts, which may lead to a left-most concept that is at low level (with inferior prediction weights), and this could deteriorate performance on the last-letter (word-completion) task.

^21^A solution for this in ANN training is training with *data augmentation*, such as injecting noise into training instances, but not all future contingencies can be anticipated at training time.

^22^Or, out of so many (random) variables, almost all needing to be 0, determine which ones should be 1 (significantly positive, i.e., relevant to the episode). One could then ask how might a system create and expand its own set of many variables.

^23^The work on neurogenesis is relevant (Nogueira et al., 2021). Others propose ways that existing “free” (unallocated) nodes can be allocated for new concepts (Valiant, 1994).
